# Incomplete Lead Removal During the Extraction Procedure: Predisposing Factors and Impact on Long-Term Survival in Infectious and Non-Infectious Cases: Analysis of 3741 Procedures

**DOI:** 10.3390/jcm12082837

**Published:** 2023-04-13

**Authors:** Andrzej Kutarski, Wojciech Jacheć, Anna Polewczyk, Dorota Nowosielecka

**Affiliations:** 1 Department of Cardiology, Medical University, 20-059 Lublin, Poland; 2 2nd Department of Cardiology, Faculty of Medical Sciences in Zabrze, Medical University of Silesia in Katowice, 41-800 Zabrze, Poland; 3 Department of Medicine and Health Sciences, The Jan Kochanowski University, 25-369 Kielce, Poland; 4 Department of Cardiac Surgery, Świętokrzyskie Center of Cardiology, 25-736 Kielce, Poland; 5 Department of Cardiology, The Pope John Paul II Province Hospital, 22-400 Zamość, Poland; 6 Department of Cardiac Surgery, The Pope John Paul II Province Hospital, 22-400 Zamość, Poland

**Keywords:** transvenous lead extraction, incomplete lead extraction, long-term outcome following lead extraction, infection of cardiac implantable electronic devices

## Abstract

Background: The long-term significance of lead remnants (LR) following transvenous lead extraction (TLE) remains disputable, especially in infectious patients. Methods: Retrospective analysis of 3741 TLEs focused on the relationship between LR and procedure complexity, complications and long-term survival. Results: The study group consisted of 156 individuals with LR (4.17%), and the control group consisted of 3585 patients with completely removed lead(s). In a multivariable model, a younger patient age at CIED implantation, more CIED procedures and procedure complexity were independent risk factors for retention of non-removable LR. Although patients with LR showed better survival outcomes following TLE (log rank *p* = 0.041 for non-infectious group and *p* = 0.017 for infectious group), multivariable Cox regression analysis did not confirm the prognostic significance of LR either in non-infectious [HR = 0.777; *p* = 0.262], infectious [HR = 0.983; *p* = 0.934] or the entire group of patients [HR = 0.858; *p* = 0.321]. Conclusions: 1. Non-removable LRs are encountered in 4.17% of patients. 2. CIED infection has no influence on retention of LRs, but younger patient age, multiple CIED-related procedures and higher levels of procedure complexity are independent risk factors for the presence of LR. 3. Better survival outcomes following TLE in patients with LRs are not the effects of their presence but younger patient and better health status.

## 1. Introduction

Transvenous lead extraction (TLE), which plays a critical role in managing patients with cardiac implantable electronic devices (CIED), is highly effective and safe provided that all safety requirements are met [1,2,3]. The goal of TLE is to remove all targeted leads in their entirety with a minimal risk of major complications [1,2,3]. Fracture of the lead during the removal procedure is not a rare complication (a few percent) [4,5,6,7,8,9,10,11], but only several papers [12,13,14] and case reports have addressed the strategy of lead fracture management [15,16,17,18,19]. Furthermore, little attention has been paid to the predictive role of incomplete lead extraction [20,21,22]. Surprisingly, there are many case reports describing lead fragments that embolize to the pulmonary arteries and the methods of their retrieval [23,24,25,26]. If possible, fragments of the broken lead should be removed [1,2,3,20,21] because they can be a source of persistent or recurrent infection after implantation of a new device [20,21]. Sometimes, the distal part of the fractured lead cannot be grasped with advanced tools as it is encased in dense scar tissue. According to the internationally agreed recommendations [1,2,3], abandonment of any lead fragment precludes procedural success; however, if the retained fragment is shorter than 4 cm (or it is just the tip of the lead), the procedure is deemed clinically successful in patients without infections. There are always doubts as to the practical application of the phrase “that does not negatively impact the outcome goals of the procedure” in infectious patients transferred to distant centres within a few days after TLE. Having a database of 3741 TLE procedures and information on patient deaths since 2006 (from the national vital statistics database), we decided to investigate the effects of retained lead fragments on long-term mortality.

The purpose of the study was to identify prognostic factors for the occurrence of lead remnants (LR) following TLE in an attempt to determine the significance of retained lead fragments in the context of long-term survival after TLE in patients with non-infectious and infectious indications for transvenous lead extraction. 

## 2. Methods

### 2.1. Study Population

All transvenous lead extraction (TLE) procedures performed between March 2006 and July 2022 at three high-volume centres were reviewed. Patient clinical data, CIED and history of pacing, information on extracted leads, procedure complexity, efficacy and outcomes were analysed using our retrospective database. The study population consisted of 3741 patients aged 5–99 years (average age 65.97 years), of which 1425 females (38.09%).

### 2.2. Lead Extraction Procedure

Indications for lead extraction, procedure effectiveness and complications were established according to the recent guidelines (2009 and 2017 HRS consensus and 2018 EHRA guidelines). The efficacy of TLE was expressed as the percentage of procedural success and clinical success [1,2,3]. 

Procedure complexity was expressed as extraction time of all leads (sheath-to-sheath time), average time of single lead extraction (sheath-to-sheath/number of extracted leads) and use of second line and advanced tools [27,28,29,30,31]. Unexpected technical problems during TLE, i.e., the situations that increased procedure complexity but caused no complications included fracture of targeted leads [4,5,6,7,8,9,10,11,12,13,14], loss of broken lead fragments [23,24,25,26], occlusion of lead implant vein in the subclavian region, Byrd dilator collapse/fracture [32], lead-on-lead binding [33], use of alternative venous access [14,33] and dislodgement of functional leads [34].

We used a stepwise approach starting with non-powered mechanical telescoping polypropylene sheaths (Byrd Dilator Sheaths, Cook Medical Inc., Bloomington, IN, USA) of all sizes and lengths. The second-line tools were powered mechanical sheath systems (Evolution Mechanical Dilator Sheaths, Cook Medical, Bloomington, IN, USA; TightRail Mechanical Rotating Dilator Sheaths, Phillips, Colorado Springs, CO, USA) or metal sheaths if the obstacle was near the venous entry site and subclavian region as previously described [13,14,15,16,19]. 

Standard stylets or locking stylets (Liberator Locking Stylets, Cook Medical Inc., Bloomington, IN, USA) were used, the latter ones for extraction of the oldest leads. Screw-out and simple traction were very rarely used because preserved or re-established venous access was important for implantation of a new or temporary pacing lead [13,14,15,16,19].

### 2.3. Extraction of Distal Fragments of the Lead

For 16 years it has been our practice to remove remnants of the lead if it was not just the lead tip. Depending on the location of the proximal end, we tried to grab the lead fragment with a lasso or basket catheter using the implant vein or subclavian access re-established after removal of another lead. Access via the jugular or femoral vein was used less frequently. The lasso or basket catheter served as an extension of the fractured lead, and the extraction procedure was continued until the lead was removed. If the distal fragments were unable to be taken out, the procedure was deemed as either a partial radiographic success or having a lack of radiographic success; thus, procedural success was dependent on lead remnant length and infection presence [13,14,15,16,19,26]. This was our working hypothesis that needed to be verified. Laser sheaths were not used.

The organization of lead extraction has evolved from procedures performed in the electrophysiology laboratory using intravenous analgesia/sedation to procedures in the hybrid room in patients only under general anaesthesia. During the last 7 years, the core extraction team has consisted of the same highly experienced extractor (now frequently serving as a proctor), experienced echocardiographer and dedicated cardiac surgeon [33,35,36].

### 2.4. Dataset and Statistical Methods

We split the case into subgroups for analysis. First, we looked at all fractures of targeted leads, which occurred in 226 patients (6.04%). Lead remnants were successfully extracted in 70 (1.87%) and remnant length was reduced (to <4 cm) in 27 (0.77%), whereas lead fragments of different lengths remained unretrievable in 117 patients (3.13%). Finally, long lead remnants (>4 cm) were found in 5 (0.13%), short lead remnants (<4 cm) in 67 (1.79%) and the lead tips in 78 (2.08%) patients, whereas 6 (0.16%) extraction procedures were not completed because of the need for emergent or planned cardiac surgery or procedure- or indication-related death in 4 (0.11%) patients. We checked the data in 5 patients with 6–7-cm lead remnants (procedure failure) and found out that 4 were non-infectious and one suffered from infective endocarditis. Four patients are alive, and one died from a non-cardiac cause.

Finally, the lead remnant group consisted of 156 patients (4.17%), including those with short lead remnants (<4 cm)—67 (1.79%), the lead tips—78 (2.08%), long lead fragments (>4 cm)—5 (0.13%), embolization of lead tips into the pulmonary circulation—5 (0.13%) and ungraspable outer silicone lead insulation in SVC in 1 (0.03%) individual. The control group consisted of 3583 patients with complete radiographic success (1132 with and 2453 without CIED infections). Five patient groups were identified: with non-infectious indications (2559), isolated pocket infection (359), infective lead-related endocarditis (823) and an all-infection group and an all-patient group. The groups were subdivided according to the presence or absence of lead remnants: LR(+) or LR(−). In each group, we compared the distribution of variables between the LR(+) and LR(−) subgroups.

**Figure 1:**
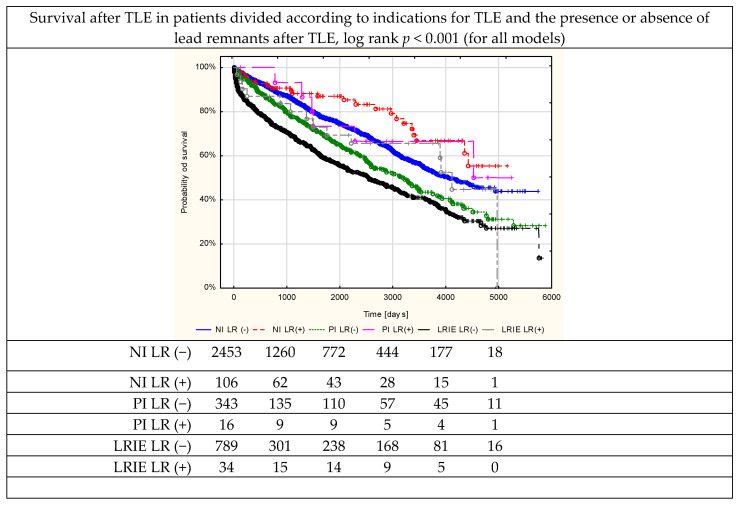

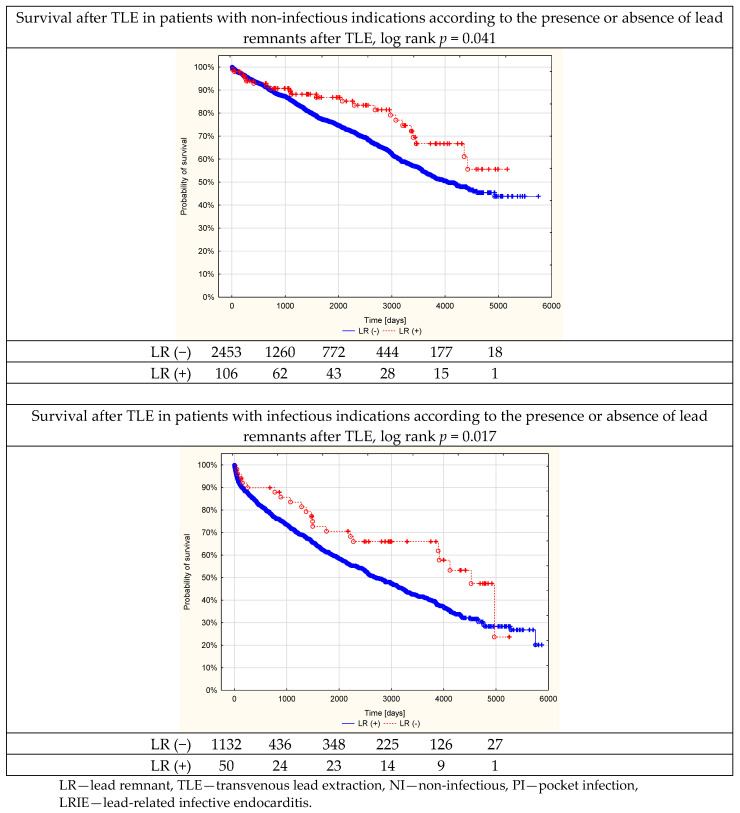
Survival after TLE according to the presence or absence of lead remnants and infectious (pocket infection or lead-related infective endocarditis) or non-infectious indications.

The clinical data, indications for lead extraction, pathogen type (Table 1), system and history of pacing, procedure-related risk factors and predictors of major complications (Table 2), as well as procedure complexity, complications, effectiveness and short- and long-term mortality (Table 3) were analysed. For uniformity, all continuous variables are presented as means ± standard deviation. The categorical variables are presented as counts and percentages. The significance of inter-group differences was determined using the nonparametric chi^2^ test with Yates correction or the unpaired Mann–Whitney U test, as appropriate. Linear regression analysis was used to evaluate prognostic factors for the occurrence of lead remnants (in the entire cohort and separately in the lead tip group and in the long remnant group). Variables with *p*-values < 0.05 under univariable analysis were entered into the multivariate model and results with a *p*-value < 0.05 under multivariable regression were presented (Table 4). To determine the impact of lead fragment retention on survival (except the first 48 h after TLE), Kaplan–Meier survival curves were plotted and evaluated with the log rank test (Figure 1). Univariable and multivariable Cox models were used to assess the factors influencing the long-term outcomes after TLE. Demographic, clinical and CIED-related data were analysed. Variables with *p*-values < 0.05 under univariable analysis were entered into the multivariate model and results with a *p*-value < 0.05 under multivariable regression were presented (Table 5). 

A *p*-value less than 0.05 was considered significant. Statistical analysis was performed using the Statistica 13.3 (TIBCO Software Inc., Palo Alto, CA, USA).

### 2.5. Approval of the Bioethics Committee

All patients gave their informed written consent to undergo TLE and use anonymous data from their medical records, approved by the Bioethics Committee at the Regional Chamber of Physicians in Lublin no. 288/2018/KB/VII. The study was carried out in accordance with the ethical standards of the 1964 Declaration of Helsinki.

## 3. Results

Retention of lead fragments (partial radiographic success) was significantly associated with younger patient age and better general health status (lack of IDD, better LVEF, less frequent heart failure and significantly lower Charlson comorbidity index). The same trend in mean and percentage differences was observed in infectious and non-infectious patients. The study groups did not differ with respect to the main pathogen type, which seemed to have no connection with the presence of lead remnants (Table 1).

It may appear that implantation of ICD and CRT-D devices “protects” against retention of lead fragments following lead extraction; however, this is due to differences in the age of the systems. Such factors as implant duration and the number of procedures before lead extraction, as well as unexpectedly higher values of the PADIT risk score, seem to be the most important predictors of lack of radiographic success after TLE in patients both with and without infections. The current study demonstrates that procedure-related risk factors for major complications and procedure complexity such as the number of extracted leads per patient, extraction of multiple leads, necessity of using alternative venous approach, extraction of abandoned lead(s) and lead dwell time were significantly associated with partial radiographic success in all study groups (Table 2). 

Table 3 summarizes the major complications of lead extraction, procedure complexity and long-term survival for prediction of complete radiographic success. Such indicators of procedure complexity as extraction time (indirectly fluoroscopy time), unexpected procedure difficulties (need to change venous approach, fracture of targeted lead, multiple difficulties) and the necessity of using second-line or advanced tools (Evolution or TightRail, metal sheath, femoral approach and lasso catheter/snare/basket catheter) were significantly more likely to be seen in infectious and non-infectious patients with partial procedural success. Major complications of TLE (any), haemopericardium and tricuspid valve damage during TLE (severe) were significantly more common in patients with incomplete lead extraction. This relationship was found in all (infectious and non-infectious) patient groups.

Mortality in the first 48 h was significantly higher in the LR(+) group (3.21% vs. 0.17%; *p* < 0.001). Surprisingly, long-term mortality during 1948 ± 1381 days of follow-up was lower in the LR(+) groups irrespective of indications for extraction (log rank *p* = 0.041 for non-infectious patients and log rank *p* = 0.017 for infectious patients) (Table 3, Figure 1).

Multivariable regression analysis showed that the number of previous CIED-related procedures and the presence of unexpected difficulties during extraction were the predictors of retention of lead fragments following TLE (for all remnants: OR = 1.323; *p* < 0.001 and OR = 4.909; *p* < 0.001, respectively). Older patient age at first CIED-related procedure and extraction of ICD lead(s) were associated with a lower probability of LR following TLE (for all remnants: OR = 0.965; *p* < 0.001 and OR = 0.393; *p* < 0.001, respectively) (Table 4).

Cox regression survival analysis confirmed the predictive value of conventional risk factors for mortality after TLE, both in the entire cohort and patients classified according to indications for lead extraction. However, we showed no effect of retained fragments on total mortality after TLE, either in the entire cohort or in the subgroups (Table 5). 

### Results Summary

Partial radiographic success was related to younger patient age and better general health status irrespective of the presence or absence of infection. Long-term mortality after TLE was independent of lead fragment retention both in non-infectious and infectious groups. Cox regression survival analysis confirmed the predictive value of traditional risk factors for mortality after TLE (both in the entire cohort and patients classified according to indications for lead extraction) and showed no influence of retained lead fragments on total mortality after TLE both in the entire cohort and in the study subgroups. 

Lead implant duration, the number of procedures before lead extraction and the PADIT risk score proved to be predictors of a lack of radiographic success after TLE in patients with and without infections. Similarly, procedure-related risk factors for major complications and procedure complexity such as the number of extracted leads per patient, extraction of multiple leads, necessity of using alternative approach, extraction of abandoned lead(s) and lead dwell time were significantly associated with partial radiographic success in all study groups. Extraction time, occurrence of unexpected procedure difficulties and the necessity of using second-line or advanced tools were significantly more common in infectious and non-infectious patients with partial procedural success. Major complications of TLE were significantly more likely to occur in patients (infectious and non-infectious) with incomplete lead extraction. The most valuable predictors of incomplete radiographic success were the number of previous CIED-related procedures and the presence of unexpected difficulties during TLE (for all remnants: OR = 1.323; *p* < 0.001 and OR = 4.909; *p* < 0.001, respectively). Older patient age at first CIED-related procedure and extraction of ICD lead(s) were associated with lower probability of LR after TLE (for all remnants: OR = 0.965; *p* < 0.001 and OR = 0.393; *p* < 0.001, respectively). 

## 4. Discussion

A considerable number of reports have been published on long-term survival following transvenous lead extraction [10,20,21,22,25,37,38,39,40,41,42,43,44]. Particularly unsatisfactory results have been reported in patients with CIED infections despite treatment in accordance with guideline recommendations [1,2,3], with mortality being 20% in the first year, 35% at 3 years and even 40–45% at five years [20,21,25,41,42,43,44,45].

The main risk factors can be classified into several categories: patient-dependent factors (heart failure, AF, low EF) [20,37,41,45], comorbidity-dependent factors (diabetes, renal failure) [20,38,39,41,42], infection-dependent factors (any infection, systemic infection, valve endocarditis, vegetations, large vegetations, vegetation remnants after TLE, MRSA infection, thrombocytopenia, anaemia) [10,25,37,38,39,40,41,43,45], CIED-related factors (any CRT infection, lead number, CRT-*p* infection) [37,41,42,43] and extraction-related factors (removal of multiple leads, lack of clinical success, major and minor extraction complications, presence of ghosts after TLE, retained lead fragments) [20,21,22,39,42,43,44].

The effects of most risk factors are obvious and most of them are beyond our control at the time of lead extraction [10,25,37,38,39,40,41,42,43,44,45]. The importance of retained lead fragments in patients with infections has been subject to discussion [20,21,22,45]. The negative impact of lead abandonment or retention of long lead fragments on long-term survival seems unquestionable [20,21,22]. On the other hand, little is known about the influence of small (<4 cm) retained fragments surrounded by scar tissue and impossible to be grasped and removed, especially in patients with infections. This study shows that leaving in place an irremovable fragment of the lead (<4 cm) or even the lead tip does not affect survival time either in non-infectious patients or in patients with various types of CIED infections. In practice, this means that when there is no possibility of long-term observation of the patient with a small lead fragment retained after TLE, clinical success can be assumed even in the case of infection.

## 5. Conclusions

(1) Non-removable lead fragments are encountered in about 4.2% of lead extraction procedures.
(2) CIED infection has no influence on partial radiographic success but younger patient age at first CIED implantation, multiple CIED-related procedures and higher complexity of lead extraction were independent risk factors for retention of non-removable lead fragments.
(3) Extraction of defibrillation (ICD) leads seems to be associated with a lower probability of retaining lead fragments.
(4) Retention of an irremovable fragment of the lead (<4 cm) or even the lead tip does not affect survival time either in non-infectious patients or in patients with various types of CIED infections.
(5) “Better survival” in patients with retained lead fragments following TLE is not a result of their presence but is related to the factors predisposing to the occurrence of remnants, especially younger patient age at first CIED implantation and better health status.

### Study Limitations

Several limitations to this study need to be considered. It presents the experience of three centres but the same first extractor. The database was prospectively integrated, but analysis was performed retrospectively. The procedures were performed using all types of mechanical systems but not laser powered sheaths. Despite a very large population of patients undergoing TLE with implant duration longer than in other studies and a similar rate of lead fracture (6%), a significant proportion of distal lead fragments were removed or their length was reduced to <4 cm. Only in five patients we were forced to leave in place a 5–7 cm fragment (lead extraction failure). The number was too small to generate a separate group for analysis (especially since four patients were still alive and one died from non-cardiac causes). The disadvantage of the study is the lack of a sufficiently large group of procedure failures; however, the technique of grasping and removing remnants, developed 17 years ago, made it possible to obtain such results.

## Figures and Tables

**Table 1:** Clinical data, indications for lead extraction and pathogen type in infectious patients.

	Non-Infectious Indications		Pocket Infection		Lead-Related Infective Endocarditis		All Infectious Indications		All Patients	
	LR(−)	LR(+)	LR(−)	LR(+)	LR(−)	LR(+)	LR(−)	LR(+)	LR(−)	LR(+)
	*n* = 2453	*n* = 106	*n* = 343	*n* = 16	*n* = 789	*n* = 34	*n* = 1132	*n* = 50	*n* = 3585	*n* = 156
	Mann–Whitney U test Chi^2^ test		Mann–Whitney U test Chi^2^ test		Mann–Whitney U test Chi^2^ test		Mann–Whitney U test Chi^2^ test		Mann–Whitney U test Chi^2^ test	
	Mean ± SD Count (%)		Mean ± SD Count (%)		Mean ± SD Count (%)		Mean ± SD Count (%)		Mean ± SD Count (%)	
Clinical data										
Patient age during TLE [years]	61.14 ± 16.00	54.62 ± 22.59 *p* < 0.001	69.87 ± 13.00	64.00 ± 15.93 *p* = 0.157	68.47 ± 13.58	64.74 ± 15.23 *p* = 0.171	68.89 ± 13.42	64.50 ± 15.29 *p* = 0.051	66.32 ± 15.33	57.79 ± 21.00 *p* < 0.001
Patient age at first CIED implantation [years]	56.59 ± 17.42	40.48 ± 23.29 *p* < 0.001	62.27 ± 13.55	52.81 ± 18.10 *p* = 0.025	60.99 ± 14.81	50.03 ± 19.26 *p* < 0.001	61.38 ± 14.44	50.88 ± 18.77 *p* < 0.001	58.10 ± 16.68	43.91 ± 22.39 *p* < 0.001
Female	1041 (42.44)	41 (38.68) *p* = 0.445	92 (26.82)	5 (31.25) *p* = 0.919	232 (29.40)	14 (41.18) *p* = 0.171	324 (28.62)	19 (38.00) *p* = 0.169	1365 (38.08)	60 (38.46) *p* = 0.876
Ischemic aetiology	1396 (56.91)	40 (37.74) *p* < 0.001	201 (58.60)	10 (62.50) *p* = 0.905	428 (54.25)	15 (44.11) *p* = 0.477	629 (55.56)	25 (50.00) *p* = 0.715	2025 (56.48)	65 (41.67) *p* < 0.001
NYHA class [I–IV]	1.83 ± 0.67	1.68 ± 0.70 *p* = 0.018	1.79 ± 0.65	1.38 ± 0.50 *p* = 0.022	1.90 ± 0.75	1.75 ± 0.73 *p* = 0.346	1.86 ± 0.72	1.63 ± 0.69 *p* = 0.047	1.84 ± 0.69	1.66 ± 0.69 *p* = 0.002
LVEF [%]	48.84 ± 15.45	54.09 ± 12.97 *p* = 0.005	49.17 ± 14.70	55.88 ± 12.18 *p* = 0.085	47.37 ± 15.04	53.86 ± 10.56 *p* = 0.007	47.83 ± 15.02	54.49 ± 11.01 *p* = 0.001	49.20 ± 15.33	54.22 ± 12.31 *p* < 0.001
Congestive heart failure (symptomatic)	480 (19.57)	15 (14.15) *p* = 0.363	55 (16.04)	0 (0.00) *p* = 0.166	153 (19.39)	1 (2.94) *p* = 0.063	208 (18.38)	1 (2.00) *p* = 0.013	688 (19.19)	16 (10.26) *p* = 0.006
Renal failure (any)	457 (18.63)	10 (9.43) *p* = 0.048	76 (22.16)	2 (12.50) *p* = 0.516	241 (30.55)	5 (14.71) *p* = 0.122	317 (28.00)	7 (14.00) *p* = 0.068	774 (21.59)	17 (10.90) *p* = 0.006
Diabetes t.2	444 (18.14)	10 (9.43) *p* = 0.022	72 (20.99)	3 (18.75) *p* = 0.852	211 (26.81)	5 (13.89) *p* = 0.118	283 (25.04)	8 (15.39) *p* = 0.157	727 (20.28)	18 (11.54) *p* = 0.008
Charlson comorbidity index [points]	4.52 ± 3.62	3.07 ± 3.20 *p* < 0.001	4.99 ± 3.46	3.94 ± 3.64 *p* = 0.136	5.49 ± 3.87	3.82 ± 3.24 *p* < 0.001	5.34 ± 3.76	3.86 ± 3.34 *p* = 0.002	4.78 ± 3.68	3.32 ± 2.26 *p* < 0.001
Indications for lead extraction										
Non-infectious	2453 (100.0)	106 (100.0) *p* = 1.000	0 (0.00)	0 (0.00)	0 (0.00)	0 (0.00)	0 (0.00)	0 (0.00)	2453 (68.42)	106 (67.95) *p* = 0.972
Infectious	0 (0.00)	0 (0.00)	343 (100.0)	16 (100.0) *p* = 1.000	789 (100.0)	34 (100.0) *p* = 1.000	1132 (100.0)	50 (100.0) *p* = 1.000	1132 (31.58)	50 (32.05) *p* = 0.783
Pocket infection (isolated)	0 (0.00)	0 (0.00)	343 (100.0)	16 (100.0) *p* = 1.000	0 (0.00)	0 (0.00)	343 (30.30)	16 (32.00) *p* = 0.938	343 (9.57)	16 (10.26) *p* = 0.935
LRIE + PI	0 (0.00)	0 (0.00)	0 (0.00)	0 (0.00)	441 (55.89)	12 (35.29) *p* = 0.014	441 (38.96)	12 (24.00) *p* = 0.032	441 (12.30)	12 (7.69) *p* = 0.103
LRIE (isolated)	0 (0.00)	0 (0.00)	0 (0.00)	0 (0.00)	348 (44.11)	22 (64.71) *p* = 0.029	348 30.74)	22 (44.00) *p* = 0.019	348 (9.71)	22 (14.10) *p* = 0.096
LRIE all	0 (0.00)	0 (0.00)	0 (0.00)	0 (0.00	789 (100.0)	34 (100.0) *p* = 1.000	789 (69.58)	34 (68.00) *p* = 0.938	789 (22.01)	34 (21.79) *p* = 0.878
Main pathogen										
Staphylococcus aureus	0 (0.00)	0 (0.00)	24 (7.96)	1 (6.67) *p* = 0.885	105 (13.89)	2 (5.88) *p* = 0.276	129 (12.08)	3 (6.12) *p* = 0.285	129 (12.01)	3 (6.12) *p* = 0.315
Staphylococcus epidermidis	0 (0.00)	0 (0.00)	63 (20.19)	3 (20.00) *p* = 0.776	196 (25.93)	7 (20.59) *p* = 0.571	259 (24.25)	10 (20.48) *p* = 0.633	259 (24.12)	10 (20.41) *p* = 0.697
Staphylococcus (other)	0 (0.00)	0 (0.00)	45 (14.42)	1 (6.67) *p* = 0.878	107 (14.15)	7 (20.59) *p* = 0.678	152 (14.23)	8 (16.33) *p* = 0.844	152 (14.15)	8 (16.33) *p* = 0.814
Other bacteria	0 (0.00)	0 (0.00)	13 (4.17)	1 (6.67) *p* = 0.863	62 (8.20)	2 (5.88) *p* = 0.956	75 (7.02)	3 (6.12) *p* = 0.876	75 (6.98)	3 (6.12) *p* = 0.829
Culture negative	0 (0.00)	0 (0.00)	112 (35.90)	7 (46.68) *p* = 0.538	175 (23.15)	11 (32.35) *p* = 0.172	287 (26.87)	18 (36.74) *p* = 0.106	292 (27.19)	18 (36.74) *p* = 0.106
Lack of culture result	0 (0.00)	0 (0.00)	55 (17.63)	2 (13.33) *p* = 0.863	111 (14.68)	5 (14.71) *p* = 0.978	166 (15.54)	7 (14.29) *p* = 0.830	167 (15.55)	7 (14.29) *p* = 0.663

LR—lead remnant, TLE—transvenous lead extraction, CIED—cardiac implantable electronic device, NYHA—New York Heart Association, LVEF—left ventricular ejection fraction, LRIE—lead-related infective endocarditis, PI—pocket infection.

**Table 2:** System and history of pacing, procedure-related risk factors and predictors of major procedure complications and complexity.

	Non-Infectious Indications		Pocket Infection		Lead-Related Infectious Endocarditis		All Infectious Indications		All Patients	
	LR(−)	LR(+)	LR(−)	LR(+)	LR(−)	LR(+)	LR(−)	LR(+)	LR(−)	LR(+)
	*n* = 2453	*n* = 106	*n* = 343	*n* = 16	*n* = 789	*n* = 34	*n* = 1132	*n* = 50	*n* = 3585	*n* = 156
	Mann–Whitney U test Chi^2^ test		Mann–Whitney U test Chi^2^ test		Mann–Whitney U test Chi^2^ test		Mann–Whitney U test Chi^2^ test		Mann–Whitney U test Chi^2^ test	
	Mean ± SD Count (%)		Mean ± SD Count (%)		Mean ± SD Count (%)		Mean ± SD Count (%)		Mean ± SD Count (%)	
System and history of pacing										
ICD—all	575 (23.44)	9 (8.49) *p* = 0.002	70 (20.41)	0 (0.00) *p* = 0.091	168 (21.29)	3 (8.82) *p* = 0.209	238 (21.03)	3 (6.00) *p* = 0.031	813 (22.68)	12 (7.69) *p* < 0.001
CRT-D pacing system	140 (5.71)	3 (2.83) *p* = 0.490	29 (8.46)	0 (0.00) *p* = 0.457	88 (11.15)	1 (2.94) *p* = 0.188	117 (10.34)	1 (2.00) *p* = 0.080	257 (7.17)	4 (2.56) *p* = 0.037
Presence of abandoned lead before TLE	202 (8.24)	23 (21.70) *p* < 0.001	45 (13.12)	3 (18.75) *p* = 0.719	127 (16.10)	13 (38.24) *p* = 0.004	172 (15.19)	16 (32.00) *p* = 0.013	374 (10.43)	39 (25.00) *p* < 0.001
Number of procedures before lead extraction	1.70 ± 0.93	2.38 ± 1.33 *p* < 0.001	2.14 ± 1.15	2.67 ± 0.82 *p* = 0.007	2.06 ± 1.23	3.24 ± 1.79 *p* < 0.001	2.09 ± 1.21	3.06 ± 1.56 *p* < 0.001	1.83 ± 1.05	2.61 ± 1.44 *p* < 0.001
PADIT score [points]	3.53 ± 2.82	4.49 ± 2.02 *p* < 0.001	3.90 ± 3.00	4.06 ± 2.57 *p* = 0.481	4.13 ± 3.02	4.79 ± 2.79 *p* = 0.186	4.06 ± 3.01	4.56 ± 2.72 *p* = 0.133	3.70 ± 2.89	4.51 ± 2.92 *p* < 0.001
Dwell time of oldest lead per patient before TLE [months]	103.4 ± 73.31	178.1 ± 94.04 *p* < 0.001	89.28 ± 68.91	134.3 ± 49.25 *p* < 0.001	89.72 ± 68.19	168.8 ± 80.43 *p* < 0.001	89.57 ± 68.40	157.8 ± 73.23 *p* < 0.001	99.04 ± 74.17	171.6 ± 88.19 *p* < 0.001
Global (cumulative) implant duration per patient [years]	15.25 ± 12.80	26.71 ± 15.24 *p* < 0.001	12.91 ± 10.80	22.57 ± 7.98 *p* < 0.001	14.39 ± 12.61	30.55 ± 15.55 *p* < 0.001	13.94 ± 12.10	28.00 ± 14.02 *p* < 0.001	14.84 ± 12.60	27.12 ± 14.82 *p* < 0.001
Procedure-related risk factors for major complications and complexity of TLE										
Number of extracted leads per patient	1.48 ± 0.64	1.75 ± 0.79 *p* < 0.001	1.88 ± 0.67	2.19 ± 0.40 *p* = 0.033	2.05 ± 0.80	2.41 ± 0.70 *p* < 0.003	2.00 ± 0.77	2.34 ± 0.63 *p* < 0.001	1.64 ± 0.72	1.94 ± 0.79 *p* < 0.001
Three or more leads extracted	152 (6.20)	17 (16.04) *p* < 0.001	44 (12.83)	16 (100.0) *p* < 0.001	164 (20.79)	14 (41.18) *p* < 0.001	208 (18.38)	17 (34.00) *p* = 0.0.10	360 (10.04)	34 (21.80) *p* < 0.001
Alternative approach	65 (2.65)	17 (16.04) *p* < 0.001	8 (2.33)	1 (6.25) *p* = 0.872	34 (4.31)	10 (29.41) *p* < 0.001	42 (2.65)	11 (22.00) *p* = 0.001	107 (2.99)	28 (17.95) *p* < 0.001
Extraction of abandoned lead(s) (any)	180 (7.34)	20 (18.87) *p* < 0.001	42 (12.25)	2 (12.50) *p* = 0.719	122 (15.460	13 (38.24) *p* = 0.001	164 (14.49)	15 (30.00) *p* = 0.005	344 (9.60)	35 (22.44) *p* < 0.001
ICD lead extraction	652 (26.58)	11 (10.38) *p* < 0.001	99 (28.86)	0 (0.00) *p* = 0.025	255 (32.32)	4 (11.77) *p* < 0.001	354 (31.27)	4 (8.00) *p* < 0.001	1006 (28.06)	15 (9.62) *p* < 0.001
Dwell time of oldest extracted lead per patient before TLE (months)	101.1 ± 75.07	177.7 ± 94.13 *p* < 0.001	87.94 ± 67.69	134.3 ± 49.25 *p* = 0.001	89.21 (67.74)	167.4 ± 78.67 *p* < 0.001	88.83 (67.70)	156.8 ± 71.79 *p* = 0.001	97.20 ± 73.04	171.0 ± 87.90 *p* < 0.001
Global (cumulative) age of extracted leads per patient (in years)	12.98 ± 12.49	23.37 ± 15.63 *p* < 0.001	12.57 ± 10.50	22.56 ± 7.98 *p* = 0.001	14.17 (12.44)	28.81 ± 14.44 *p* < 0.001	13.69 ± 11.90	26.81 ± 12.98 *p* = 0.001	13.21 ± 12.31	25.15 ± 14.83 *p* < 0.001

LR—lead remnant, TLE—transvenous lead extraction, ICD—cardiac implantable cardioverter defibrillator, CRT-D—cardiac resynchronisation therapy cardioverter defibrillator.

**Table 3:** TLE complexity, complications, effectiveness and short- and long-term mortality.

	Non-Infectious Indications		Pocket Infection		Lead-Related Infectious Endocarditis		All Infectious Indications		All Patients	
	LR(−)	LR(+)	LR(−)	LR(+)	LR(−)	LR(+)	LR(−)	LR(+)	LR(−)	LR(+)
	*n* = 2453	*n* = 106	*n* = 343	*n* = 16	*n* = 789	*n* = 34	*n* = 1132	*n* = 50	*n* = 3585	*n* = 156
	Mann–Whitney U test Chi^2^ test		Mann–Whitney U test Chi^2^ test		Mann–Whitney U test Chi^2^ test		Mann–Whitney U test Chi^2^ test		Mann–Whitney U test Chi^2^ test	
	Mean ± SD Count (%)		Mean ± SD Count (%)		Mean ± SD Count (%)		Mean ± SD Count (%)		Mean ± SD Count (%)	
Procedure complexity and outcomes										
Procedure duration (sheath-to-sheath) [minutes]	13.08 ± 18.41	50.13 ± 62.44 *p* < 0.001	13.07 ± 19.05	26.00 ± 22.00 *p* < 0.001	16.02 ± 23.38	36.71 ± 31.05 *p* < 0.001	15.12 ± 22.19	33.28 ± 28.68 *p* < 0.001	13.73 ± 19.70	44.73 ± 54.44 *p* < 0.001
Average time of single lead extraction * [minutes]	8.70 ± 11.62	28.38 ± 32.81 *p* < 0.001	6.87 ± 8.91	12.07 ± 10.20 *p* = 0.009	7.51 ± 9.33	15.11 ± 11.83 *p* < 0.001	7.32 ± 9.20	14.14 ± 11.32 *p* < 0.001	8.26 ± 10.93	23.76 ± 28.47 *p* < 0.001
Unexpected procedure difficulty (any)	460 (18.75)	69 (65.09) *p* < 0.001	42 (12.54)	10 (62.50) *p* < 0.001	109 (13.82)	18 (52.94) *p* < 0.001	151 (13.34)	28 (56.00) *p* < 0.001	611 (17.04)	97 (62.18) *p* < 0.001
Need to change venous approach	57 (2.32)	17 (16.04) *p* < 0.001	8 (2.33)	2 (12.50) *p* = 0.101	33 (4.18)	10 (29.41) *p* < 0.001	41 (3.62)	12 (24.00) *p* < 0.001	98 (2.73)	29 (18.95) *p* < 0.001
Fracture of extracted lead	57 (2.32)	45 (42.45) *p* < 0.001	7 (2.04)	7 (43.75) *p* < 0.001	15 (1.90)	13 (38.24) *p* < 0.001	22 (1.94)	20 (40.00) *p* < 0.001	79 (2.20)	65 (41.67) *p* < 0.001
Two or more technical problems	122 (4.79)	47 (44.34) *p* < 0.001	15 (4.37)	5 (32.25) *p* < 0.001	35 (4.44)	13 (38.24) *p* < 0.001	50 (4.42)	18 (36.00) *p* < 0.001	172 (4.80)	65 (41.67) *p* < 0.001
Use of second line/advanced tools										
Evolution (old and new) or TightRail	28 (1.14)	14 (13.21) *p* < 0.001	4 (1.17)	1 (6.25) *p* = 0.545	6 (0.76)	2 (5.88) *p* = 0.046	10 (0.88)	3 (6.00) *p* = 0.010	38 (1.06)	17 (10.90) *p* < 0.001
Metal sheath	209 (8.52)	32 (30.19) *p* < 0.001	17 (4.96)	2 (12.50) *p* = 0.455	41 (5.20)	3 (8.82) *p* = 0.664	58 (5.12)	5 (10.00) *p* = 0.278	267 (7.45)	37 (23.72) *p* < 0.001
Lasso catheter/snare/basket catheter	96 (3.92)	36 (33.96) *p* < 0.001	7 (2.04)	6 (37.50) *p* < 0.001	26 (3.30)	9 (26.47) *p* < 0.001	33 (2.92)	15 (30.00) *p* < 0.001	129 (3.60)	51 (32.69) *p* < 0.001
TLE efficacy and complications										
Major complications (any)	46 (1.88)	10 (9.43) *p* < 0.001	3 (0.88)	2 (12.50) *p* = 0.005	12 (1.52)	4 (11.77) *p* < 0.001	15 (1.33)	6 (12.00) *p* < 0.001	61 (1.70)	16 (10.26) *p* < 0.001
Haemopericardium	30 (1.22)	8 (7.55) *p* = 0.002	2 (0.58)	1 (6.25) *p* = 0.303	6 (0.76)	3 (8.82) *p* < 0.001	7 (0.62)	2 (4.00) *p* < 0.001	36 (1.00)	12 (7.69) *p* < 0.001
Tricuspid valve damage during TLE (severe)	14 (0.57)	2 (1.89) *p* = 0.308	0 (0.00)	1 (6.25) *p* = 0.027	5 (0.63)	0 (0.00) *p* = 0.508	5 (0.44)	1 (2.00) *p* = 0.639	19 (0.53)	3 (1.92) *p* = 0.095
Emergent cardiac surgery	25 (1.02)	7 (6.60) *p* = 0.042	2 (0.58)	1 (6.25) *p* = 0.159	5 (0.63)	3 (8.82) *p* < 0.001	7 (0.62)	3 (6.00) *p* = 0.002	32 (0.893)	10 (6.41) *p* < 0.001
Complete clinical success	2436 (99.31)	101 (95.28) *p* < 0.001	342 (99.71)	1 (6.25) *p* < 0.001	779 (98.73)	4 (11.77) *p* < 0.001	1121 (99.03)	5 (10.00) *p* < 0.001	3557 (99.22)	105 (67.31) *p* < 0.001
Complete procedural success	2436 (99.31)	0 (0.00) *p* < 0.001	342 (99.71)	0 (0.00) *p* < 0.001	779 (98.73)	1 (2.94) *p* < 0.001	1121 (99.03)	1 (2.00) *p* < 0.001	3557 (99.22)	1 (0.65) *p* < 0.001
Death, procedure related (intra-, post-procedural)	3 (0.12)	2 (1.89) *p* = 0.004	0 (0.00)	0 (0.00)	0 (0.00)	1 (2.94) *p* = 0.021	0 (0.00)	1 (2.00) *p* = 0.023	3 (0.08)	3 (1.92) *p* < 0.001
Death, indication-related (intra-, post-procedural)	0 (0.00)	0 (0.00)	1 (0.29)	0 (0.00) *p* = 0.835	2 (0.25)	1 (2.94) *p* = 0.274	3 (0.27)	1 (2.00) *p* = 0.410	3 (0.08)	1 (0.64) *p* = 0.404
Survival at follow-up										
Log rank *p* for all models	*p* < 0.001									
Survivors at follow-up	1743 (71.06)	82 (77.34)	181 (52.77)	10 (62.50)	354 (44.87)	17 (50.00)	535 (47.26)	27 (54.00)	2278 (63.54)	109 (69.87)
<First 2 days after TLE	3 (0.12)	1 (0.94) *p* = 0.404	1 (0.29)	0 (0.00) *p* = 0.829	2 (0.25)	4 (11.76) *p* < 0.001	3 (0.27)	4 (8.00) *p* < 0.001	6 (0.17)	5 (3.21) *p* < 0.001
One-month mortality after TLE	18 (0.73)	2 (1.89) *p* = 0.455	4 (1.17)	0 (0.00) *p* = 0.433	35 (4.44)	4 (11.76) *p* = 0.120	39 (3.45)	4 (8.00) *p* = 0.196	57 (1.59)	6 (3.85) *p* = 0.068
One-year mortality after TLE	131 (5.34)	7 (6.60) Log rank *p* = 0.811	28 (8.16)	0 (0.00) Log rank *p* = 0.244	140 (17.74)	7 (20.59) Log rank *p* = 0.418	168 (14.84)	7 (14.00) Log rank *p* = 0.208	299 (8.34)	14 (8.97) Log rank *p* = 0.461
Three-year mortality after TLE	211 (8.60)	11 (10.38) Log rank *p* = 0.419	71 (20.70)	1 (6.25) Log rank *p* = 0.132	237 (30.04)	9 (26.47) Log rank *p* = 0.158	308 (27.21)	10 (20.00) Log rank *p* = 0.046	619 (17.27)	21 (13.46) Log rank *p* = 0.054
Total mortality at follow-up	710 (28.94)	24 (22.64) Log rank *p* = 0.012	162 (47.23)	6 (37.50) Log rank *p* = 0.181	435 (55.13)	17 (50.00) Log rank *p* = 0.052	597 (52.74)	23 (46.00) Log rank *p* = 0.010	1307 (36.46)	47 (30.13) Log rank *p* = 0.041

LR—lead remnant, TLE—transvenous lead extraction.

**Table 4:** Predictive factors for retention of lead fragments after TLE in univariable and multivariable regression analysis.

	Univariable Linear Regression			Multivariable Linear Regression		
	OR	95% CI	*p*	OR	95% CI	*p*
The tip of the lead left after TLE						
Patient age at first CIED implantation [by 1 year]	0.977	0.966–0.989	*p* < 0.001	0.984	0.972–0.997	*p* = 0.013
Number of procedures before lead extraction [by 1]	1.498	1.285–1.747	*p* < 0.001	1.362	1.153–1.609	*p* < 0.001
Unexpected procedure difficulty (any) [y/n]	3.027	1.856–4.973	*p* < 0.001	2.211	1.327–3.686	*p* = 0.002
Lead or longer fragment of the lead retained after TLE						
Patient age at first CIED implantation [by 1 year]	0.964	0.955–0.974	*p* < 0.001	0.978	0.967–0.988	*p* < 0.001
ICD lead extraction [y/n]	0.231	0.106–0.502	*p* < 0.001	0.311	0.141–0.685	*p* = 0.004
Number of procedures before lead extraction [by 1]	1.628	1.420–1.868	*p* < 0.001	1.330	1.134–1.560	*p* < 0.001
Unexpected procedure difficulty (any) [y/n]	9.815	6.192–15.56	*p* < 0.001	6.319	3.903–10.23	*p* < 0.001
All remnants present after TLE						
Patient age at first CIED implantation [by 1 year]	0.965	0.958–0.972	*p* < 0.001	0.975	0.968–0.983	*p* < 0.001
ICD lead extraction [y/n]	0.303	0.182–0.503	*p* < 0.001	0.393	0.234–0.662	*p* < 0.001
Number of procedures before lead extraction [by 1]	1.607	1.444–1.789	*p* < 0.001	1.323	1.171–1.494	*p* < 0.001
Unexpected procedure difficulty (any) [y/n]	7.483	5.392–10.38	*p* < 0.001	4.909	3.478–6.927	*p* < 0.001

CIED—cardiac implantable electronic device, TLE—transvenous lead extraction, ICD—cardiac implantable cardioverter defibrillator.

**Table 5:** Prognostic factors for survival after TLE in the entire cohort of patients in univariable and multivariable Cox regression analysis.

	Univariable Cox Regression			Multivariable Cox Regression		
Entire Cohort						
	HR	95% CI	*p*	HR	95% CI	*p*
Patient age during TLE [by 1 year]	1.050	1.045–1.055	*p* < 0.001	1.046	1.040–1.052	*p* < 0.001
Female [y/n]	0.653	0.582–0.733	*p* < 0.001	0.927	0.817–1.051	*p* = 0.238
Ischemic aetiology [y/n]	1.799	1.610–2.012	*p* < 0.001	0.950	0.852–1.080	*p* = 0.491
NYHA functional class [by 1]	2.149	2.000–2.308	*p* < 0.001	1.345	1.224–1.477	*p* < 0.001
LVEF [by 1 %*p*]	0.966	0.962–0.969	*p* < 0.001	0.979	0.974–0.984	*p* < 0.001
Renal failure (any) [y/n]	3.245	2.896–3.635	*p* < 0.001	1.835	1.628–2.068	*p* < 0.001
Diabetes t. 2 [y/n]	1.825	1.616–2.061	*p* < 0.001	1.322	1.168–2.068	*p* < 0.001
Charlson comorbidity index [by 1 point]	1.146	1.131–1.161	*p* < 0.001			
Pocket infection [y/n]	1.178	1.001–1.386	*p* = 0.048	1.300	1.095–1.545	*p* = 0.001
Lead-related infective endocarditis [y/n]	1.717	1.532–1.924	*p* < 0.001	1.614	1.430–1.822	*p* < 0.001
ICD VR-DR before TLE [y/n]	1.182	1.043–1.340	*p* = 0.009	1.126	0.969–1.308	*p* = 0.123
CRTD before TLE [y/n]	2.274	1.905–2.716	*p* < 0.001	1.308	1.071–1.598	*p* = 0.009
^1^ Lead remnant after TLE [y/n]	0.622	0.460–0.840	*p* = 0.002	0.858	0.634–1.161	*p* = 0.321
Non-infectious indications for lead extraction						
Patient age during TLE [by 1 year]	1.053	1.046–1.060	*p* < 0.001	1.052	1.043–1.061	0.000
Female [y/n]	0.632	0.543–0.735	*p* < 0.001	0.877	0.738–1.043	0.137
Ischemic aetiology [y/n]	2.027	1.738–2.364	*p* < 0.001	0.921	0.779–1.089	0.334
NYHA functional class [by 1]	2.246	2.034–2.480	*p* < 0.001	1.262	1.103–1.443	0.001
LVEF [by 1 %*p*]	0.964	0.959–0.968	*p* < 0.001	0.977	0.971–0.984	0.000
Renal failure (any) [y/n]	3.419	2.924–3.997	*p* < 0.001	1.787	1.511–2.113	0.000
Diabetes t. 2 [y/n]	1.773	1.497–2.099	*p* < 0.001	1.242	1.043–1.478	0.015
Charlson comorbidity index [by 1 point]	1.146	1.126–1.167	*p* < 0.001			
ICD VR-DR before TLE [y/n]	1.166	1.001–1.374	*p* = 0.049	1.187	0.982–1.437	0.077
CRTD before TLE [y/n]	2.084	1.594–2.723	*p* < 0.001	1.258	0.942–1.681	0.120
^2^ Lead remnant after TLE [y/n]	0.643	0.429–0.967	*p* = 0.034	0.983	0.653–1.479	0.934
Infectious indications for lead extraction						
Patient age during TLE [by 1 year]	1.042	1.034–1.049	*p* < 0.001	1.039	1.030–1.048	*p* < 0.001
Female [y/n]	0.833	0.696–0.997	*p* = 0.047	0.971	0.804–1.173	*p* = 0.760
Ischemic aetiology [y/n]	1.572	1.337–1.852	*p* < 0.001	0.982	0.826–1.168	*p* = 0.842
NYHA functional class [by 1]	2.013	1.818–2.229	*p* < 0.001	1.466	1.283–1.674	*p* < 0.001
LVEF [by 1 %*p*]	0.968	0.963–0.973	*p* < 0.001	0.981	0.973–0.989	*p* < 0.001
Renal failure (any) [y/n]	2.751	2.331–3.248	*p* < 0.001	1.881	1.583–2.235	*p* < 0.001
Diabetes t. 2 [y/n]	1.727	1.449–2.060	*p* < 0.001	1.407	1.178–1.682	*p* < 0.001
Charlson comorbidity index [by 1 point]	1.135	1.112–1.157	*p* < 0.001			
ICD VR-DR before TLE [y/n]	1.332	1.096–1.620	*p* = 0.004	0.981	0.763–1.260	*p* = 0.878
CRTD before TLE [y/n]	2.170	1.711–2.751	*p* < 0.001	1.269	0.948–1.698	*p* = 0.109
^3^ Lead remnant after TLE y/n]	0.605	0.391–0.935	*p* = 0.024	0.777	0.499–1.208	*p* = 0.262
Staphylococcus aureus [y/n]	1.324	1.051–1.667	*p* = 0.017	1.211	0.950–1.542	*p* = 0.122
Staphylococcus epidermidis [y/n]	0.816	0.677–0.983	*p* = 0.032	0.885	0.725–1.081	*p* = 0.231
Other Staphylococci [y/n]	1.084	0.626–1.878	*p* = 0.774			
Other bacteria [y/n]	1.760	1.331–2.328	*p* < 0.001	1.463	1.093–1.960	*p* = 0.011
Culture negative [y/n]	0.857	0.712–1.033	*p* = 0.105			
Lack of culture results [y/n]	1.137	0.907–1.426	*p* = 0.265			

^1^ Lead remnant after TLE—variant of multivariable model: (the lead tip); [HR = 0.909: 95% CI (0.596–1.388); *p* = 0.660], (longer portion of the lead); [HR = 0.839: 95% CI (0.538–1.311); *p* = 0.441]. ^2^ Lead remnant after TLE—variant of multivariable model: (the lead tip); [HR = 0.973: 95% CI (0.549–1.724); *p* = 0.926], (longer portion of the lead); [HR = 0.999: 95% CI (0.562–1.776); *p* = 0.997]. ^3^ Lead remnant after TLE—variant of multivariable model: (the lead tip); [HR = 0.806: 95% CI (0.430–1.513); *p* = 0.502], (longer portion of the lead); [HR = 0.810: 95% CI (0.413–1.586); *p* = 0.537]. TLE—transvenous lead extraction; NYHA class—New York Heart Association functional class; LVEF—left ventricular ejection fraction; ICD—implantable cardioverter defibrillator—single chamber (VR) or dual chamber (DR); CRT-D—cardiac resynchronisation therapy cardioverter defibrillator.

## Data Availability

Readers can access the data supporting the conclusions of the study at www.usuwanieelektrod.pl, accessed on 1 August 2021.
